# Supplementary figures and images for: Dissection of flag leaf metabolic shifts and their relationship with those occurring simultaneously in developing seed by application of non-targeted metabolomics
Source: PLoS One. 2020 Jan 24;15(1):e0227577. doi: 10.1371/journal.pone.0227577 (PMC6980602; doi:10.1371/journal.pone.0227577)

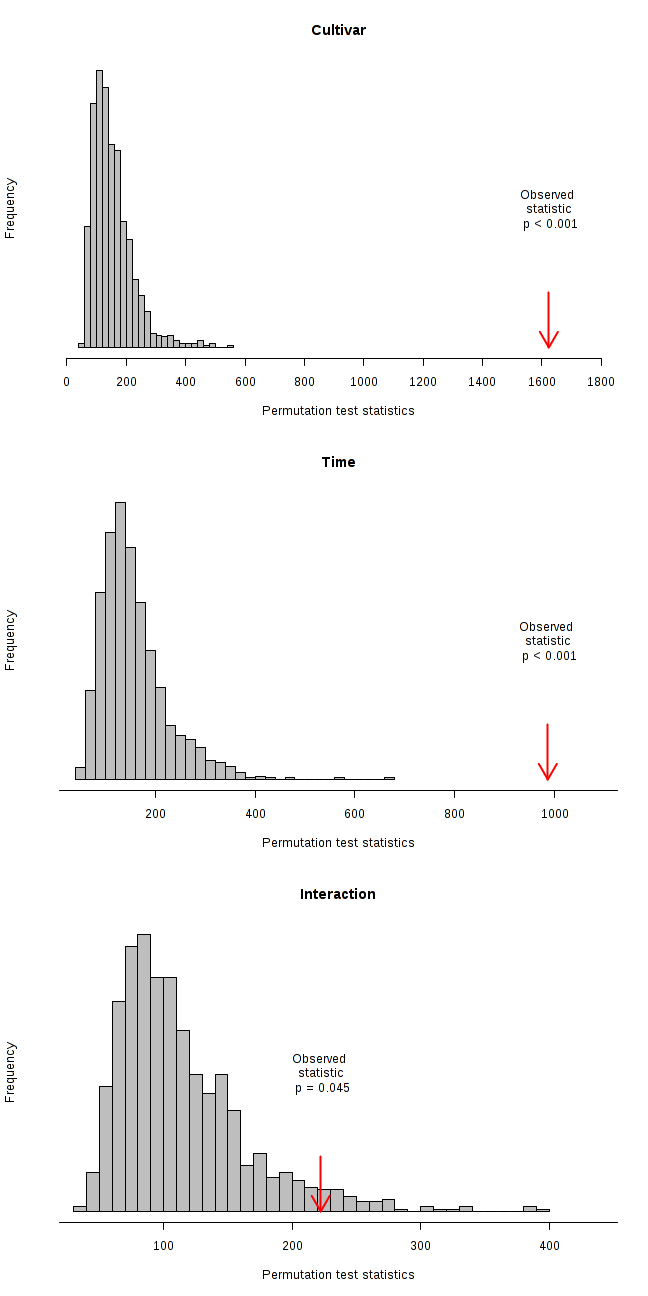


Figure S1. The results of model validations through permutations

Supplement: S1 Fig — (DOCX) [file pone.0227577.s001.docx]
