# Supplementary material for: Dissection of flag leaf metabolic shifts and their relationship with those occurring simultaneously in developing seed by application of non-targeted metabolomics
Source: PLoS One. 2020 Jan 24;15(1):e0227577. doi: 10.1371/journal.pone.0227577 (PMC6980602; doi:10.1371/journal.pone.0227577)
Supplement: S2 Fig — (DOCX) [file pone.0227577.s002.docx]

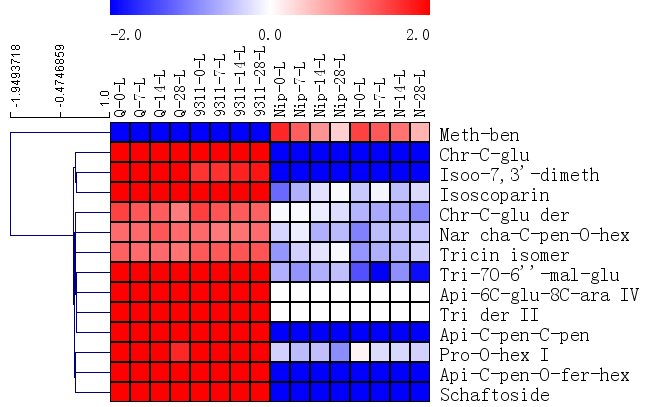


Figure S2. Heatmap of the metabolites that stood out based on the major pattern of cultivar. Q, Nip and N represent Qingfengai, Nipponbare and Nongken 58, respectively. Ratios of fold changes are given by shades of red or blue colors according to the scale bar. Data represent mean values of four biological replicates for each cultivar and time point. For full metabolite names, refer to Table S2.
