# Supplementary material for: Dissection of flag leaf metabolic shifts and their relationship with those occurring simultaneously in developing seed by application of non-targeted metabolomics
Source: PLoS One. 2020 Jan 24;15(1):e0227577. doi: 10.1371/journal.pone.0227577 (PMC6980602; doi:10.1371/journal.pone.0227577)
Supplement: S4 Fig — (A) The LC-MS total ion chromatograph (positive ion mode) of flag leaf and developing seed of Qingfengai at 7 DAF. (B) PCA score plot of the metabolomes of rice flag leaves and developing seeds. (DOCX) [file pone.0227577.s004.docx]

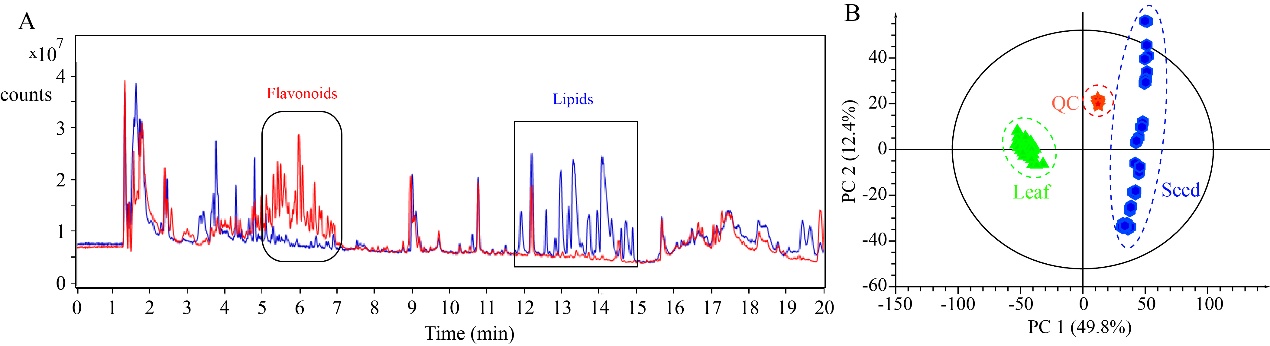


Figure S4. Different metabolomes of rice flag leaf and developing seed. (A) The LC-MS total ion chromatograph (positive ion mode) of flag leaf and developing seed of Qingfengai at 7 DAF. (B) Principal component analysis of the metabolomes of rice flag leaves and developing seeds.
